# Supplementary material for: Content-rich biological network constructed by mining PubMed abstracts
Source: BMC Bioinformatics. 2004 Oct 8;5:147. doi: 10.1186/1471-2105-5-147 (PMC528731; doi:10.1186/1471-2105-5-147)
Supplement: Additional File 2 — The original results of the above study (non-essential files are deleted to keep the file size under the limit set by BMC bioinformatics). [file 1471-2105-5-147-S2.bz2 › chilibotAdditionalFile2/dip05/205ID8749394E700/html/SUH_NOTCH1.html]

 


 **SUH** and **NOTCH1** 
  
Found 2 abstracts in PubMed, retrieved 2.  
 

 What does Google say? 
 PDF only 
| .edu only 

---

**Interactive relationship** (e.g. stimulation, inhibition, etc)

**Non-interactive relationship** (e.g. studied together, co-existance, homology, etc.)

- The adhesive effects of Delta1 and Delta T were independent of  **SuH**  dependent Notch  [ **NOTCH1** ]  signalling.  Ref: 11520669 Mech Dev, 2001
- However, nonsegmental expression of fak mRNA occurs with ectopic activation of Notch  [ **NOTCH1** ]  signaling through  **SuH**  and also in fused somite and beamter mutant embryos, indicating that there are multiple regulators of fak mRNA expression.  Ref: 11784077 Dev Biol, 2001
- In addition, we show that neither Notch  [ **NOTCH1** ]  signaling through Suppressor of Hairless  **SuH**  nor deltaD is necessary for the wild type segmental pattern of fak mRNA expression in the anterior paraxial mesoderm.  Ref: 11784077 Dev Biol, 2001
